# Expectations of Patients and Physicians Regarding Patient-Accessible Medical Records

**DOI:** 10.2196/jmir.7.2.e13

**Published:** 2005-05-24

**Authors:** Stephen E Ross, Jamie Todd, Laurie A Moore, Brenda L Beaty, Loretta Wittevrongel, Chen-Tan Lin

**Affiliations:** ^2^Colorado Health Outcomes ProgramAurora, COUSA; ^1^Division of General Internal MedicineUniversity of Colorado Health Sciences CenterAurora, COUSA

**Keywords:** Patient access to records, medical records systems, computerized, questionnaires, physician-patient relations, medical records

## Abstract

**Background:**

Middle class populations have supported shared medical records, including Internet-accessible medical records. The attitudes of lower income populations, and of physicians, are less clear.

**Objectives:**

The objective of this study was to compare the attitudes toward shared outpatient medical records among (1) socioeconomically disadvantaged patients in community health centers, (2) insured patients in primary care offices, and (3) a broad range of physicians in outpatient practice.

**Methods:**

Written questionnaires were provided to patients in the waiting rooms of six primary care practices in the metropolitan Denver, Colorado area. Three practices were community health centers, and three practices were primary care clinics of an academic medical center. Questionnaires were also mailed to primary care physicians in the state of Colorado.

**Results:**

There was a 79% response rate for patient surveys (601 surveys returned) and a 53% response rate for physician surveys (564 surveys returned). Academic medical center patients and community health center patients were equally likely to endorse shared medical records (94% vs 96%) and Internet-accessible records (54% vs 57%). Community health center patients were more likely than academic medical center patients to anticipate the benefits of shared medical records (mean number of expected benefits = 7.9 vs 7.1, *P* < .001), and they were also somewhat more likely to anticipate problems with shared records. Significant predictors of patient endorsement of Internet-accessible records were previous use of the Internet (OR = 2.45, CI 1.59–3.79), the number of expected benefits (OR = 1.12 per unit, CI 1.03–1.21), anticipation of asking more questions between visits (OR = 1.73, CI 1.18–2.54), and anticipation of finding the doctors' notes to be confusing (OR = 1.50, CI 1.01–2.22). Physicians were significantly more likely than patients to anticipate that access to records would cause problems. Physicians were significantly less likely than patients to anticipate benefits (mean number of expected benefits = 4.2 vs 7.5, *P* < .001).

**Conclusions:**

Interest in shared medical records is not confined to a white, middle class population. Shared medical records are almost universally endorsed across a broad range of ethnic and socioeconomic groups. A majority of patients are also interested in Internet-accessible records, but a substantial minority is not. The primary determinants of support of Internet-accessible records are not age, race, or education level; rather, they are previous experience with the Internet and patients' expectations of the benefits and drawbacks of reading their medical records. Physicians have more concerns about shared medical records and see less potential for benefit. The attitudes of patients and physicians may need to be reconciled for widespread adoption of shared medial records to be achieved.

## Introduction

As the Health Insurance Portability and Accountability Act (HIPAA) has clarified the rights of patients to review their medical records [1], there has been increasing interest in sharing records with patients, particularly in an online format [2-6]. These clinical trials and other studies of shared paper records [7] have suggested that patient-accessible medical records may improve doctor-patient communication, patient adherence to treatment, patient education, and patient empowerment, all with little risk. Nonetheless, concerns remain, particularly among physicians, that patient-accessible medical records might increase physician workload or disrupt the doctor-patient relationship [8].

Several recent surveys have evaluated patients' interest in shared records. A study of patients in Minnesota found that 79% of patients were “very interested” or “somewhat interested” in reading their clinic medical record [9]. Roughly half were interested in reading a paper copy of their medical record at home, and roughly half were interested in an online version. The authors noted a “strong polarity” of opinion about the latter, with one patient threatening to sue if records were made available online. A study of patients in the United Kingdom had similar findings, with 83% of patients endorsing of patient-accessible records and roughly half expressing interest in viewing records using a computer [10]. It remains unclear to what extent this interest in shared medical records currently extends to patients of lower socioeconomic status in the United States, particularly those in “safety-net” medical programs. Similarly, although physician attitudes towards shared records have been assessed in small samples [11-13], broad attitudes of practicing physicians remain undefined.

We addressed these issues through two related survey projects. In one project, we assessed the attitudes of a broad sample of physicians in the state of Colorado using a mailed questionnaire. In a follow-up project, we assessed patient attitudes in multiple primary care offices in the metropolitan Denver area. Half of these offices were associated with a community health center for socioeconomically disadvantaged patients, and half were primary care clinics of an academic medical center that provided services for a more middle class clientele. Our objectives were to compare the attitudes of patients in the two groups and to compare the attitudes of patients as a whole to those of doctors in the region.

## Methods

### Questionnaire Design

Physician and patient questionnaires included demographic items and 16 questions assessing the potential benefits and concerns of sharing medical records (Multimedia Appendix). Key themes were identified from a review of previous studies of patient-accessible medical records [7]. Most of the questions had been used before in a clinical trial of patient-accessible medical records [6]. In that study, pilot testing was performed one-on-one with patients to ensure comprehensibility and lack of ambiguity in the questions.

Patients also answered two additional questions regarding their attitudes about shared medical records in general, and two additional questions regarding shared *online* medical records. As the primary intent of the survey was to assess attitudes towards shared medical records regardless of format, the latter two questions were the only ones in either survey to mention online medical records. Both the patient and physician surveys were approved by the Colorado Multiple Institutional Review Board.

### Patient Survey

The survey population represented outpatients to primary care practices in metropolitan Denver, Colorado. The sample frame consisted of adult patients (18 years of age and older) presenting for outpatient appointments to one of six primary care sites between September 1, 2003 and April 27, 2004. Three primary care practices associated with a teaching hospital (University of Colorado Hospital) represented patients typical of a private practice. Three neighborhood community health centers associated with the safety-net hospital (Denver Health) represented a financially disadvantaged and ethnically diverse population. A convenience sample was obtained from patients in the waiting rooms of these practices. All patients with appointments were potentially eligible. Because the medical records were written in English and we intended to study the attitudes of patients who would be reading their own medical records, patients who did not speak English were not approached for the survey.

Questionnaires were given to patients by a research assistant stationed in the waiting rooms of the practices. Surveys were anonymous, but the research assistant tracked how many patients declined to complete the survey. Surveys were abstracted and double-entry verified.

### Physician Survey

The survey of physicians was performed in July 2002. The survey population represented Colorado physicians in primary care (family practice, general internal medicine, and general practice) and in internal medicine specialties. The sample frame was derived from a list of Colorado physicians supplied by the Colorado Commission on Family Medicine. The original sample frame contained 4351 physician records with information on degree, specialty, age, gender, and street address. The database was cleaned to eliminate specialties not of interest to this study (615), duplicate entries (417), and entries without the full complement of information (50 due to missing age information, 6 due to missing gender information). This resulted in a cleaned database containing 3263 records. A probability sample was created by randomly selecting one fourth of the physicians in the primary care group and one half of the physicians in the internal medicine specialty group. This produced a sample of 1059 physicians, 580 in primary care and 479 in internal medicine specialties.

Questionnaires were mailed to physicians in July 2002. Potential respondents were initially mailed a postcard describing the survey. A written questionnaire was mailed one week later with a business reply envelope. A reminder card was sent two weeks later. A second questionnaire was mailed to those who did not respond within four weeks.

### Statistical Methods

Statistical analyses were performed using SAS Version 9.1 (SAS Institute, Cary, NC). Differences in dichotomous outcomes were compared using chi-square tests, and differences in continuous outcomes were compared using *t* tests. Internal consistency was evaluated by Cronbach alpha. Logistic regression was used for multivariate analysis. All tests were considered significant at the 0.05 level. Because the proportion of missing values was less than 5% for every questionnaire item, we did not incorporate adjustment or imputation for missing values in the multivariate analysis.

## Results

### Sample Size and Response Rate

For patients, 601 surveys were returned, 295 from the community health centers (response rate 71%) and 306 from the academic primary care clinics (response rate 88%). For physicians, 340 questionnaires were returned from the primary care group (response rate 59%) and 224 from the specialist group (response rate 47%).

### Demographics

The majority of respondents in both patient groups were female, with a mean age in the 40s (Table 1). Twenty-one percent of the patients were African American, and 13% were Hispanic. Patients in the community health center were less likely to be white, non-Hispanic, and they had a lower socioeconomic status than those in the academic primary care clinics. Although patients in the community health center were less likely to have Internet access at home or work, half of them did have such access, and the majority of patients in both patient groups had used the Internet before. A substantial minority of patients in the community health center (48%) and a majority of patients in the academic primary care clinics (63%) answered “yes” to “Have you reviewed parts of your medical records before?”

**Table 1:** Patient demographics

	**Community** **Health Center Patients** **(n = 306)** **No. (%)**	**Academic Primary Care Clinic Patients** **(n = 295)** **No. (%)**	***P* value**
Age (years), mean (SD)	42 (15)	49 (18)	< .001
Male gender	75 (28)	108 (37)	.02
White, non-Hispanic	95 (35)	222 (75)	< .001
Household income > $45000 per year	16 (6)	145 (52)	< .001
College graduate	53 (20)	165 (56)	< .001
Insurance other than Medicaid, Medically Indigent, or uninsured	57 (22)	263 (89)	< .001
More than three physician visits per year	120 (41)	95 (31)	.01
Used Internet before	182 (67)	241 (82)	< .001
Have Internet access at home or work	148 (54)	242 (83)	< .001
Interested in communicating with doctor by email	129 (48)	190 (66)	< .001
Reviewed parts of their medical records before	131 (48)	187 (63)	< .001

For physicians, the age and gender distribution of the respondent sample was representative of the sample frame (Table 2). The mean age for the respondent sample was within one year of the overall group, and the percentage of males in the respondent sample was within 1% of the overall group.

**Table 2:** Physician demographics

	**All Physicians** **(N = 564)** **No. (%)**
Age (years), mean (SD)	48 (10)
Male	421 (75)
Office-based practice	535 (97)
Already routinely send notes to patients	45 (8)

### Patient Attitudes in the Two Settings

The responses of patients in the community health centers were compared with those from patients in the academic primary care clinics. Because the responses to the nine questions about potential benefits of access to the medical record were highly correlated (Cronbach alpha = 0.90), the count of the number of questions which were answered “strongly agree” or “agree” was created, which we termed the number of expected benefits.

In general, the patients in the community health centers (CHCs) were more likely to anticipate benefits (Table 3), but they were also more likely to anticipate encountering difficulties with shared records (Table 4). The number of expected benefits was high in both patient groups, modestly higher in the CHC patients. The CHC patients were particularly more likely to anticipate that they would better understand their doctors' instructions, better adhere to their doctors' recommendations, and feel more in control of their medical care. These positive expectations were noted in spite of the fact that the CHC patients were also more likely to anticipate being confused by various parts of the medical record and being embarrassed or offended by the doctors' notes.

Patients were also asked two summary questions about shared records, in general, and about shared records online, in particular. Ninety-five percent of all patients agreed with the statement, “Overall, I think it is a good idea for patients to be able to routinely review their outpatient medical records” (96% of CHC patients vs 94% of academic primary care clinic patients, *P* = .31). Fifty-six percent of all patients agreed with the statement, “Overall, I think it is a good idea for patients to be able to review their outpatient medical records using the Internet” (57% of CHC patients vs 54% of academic primary care clinic patients, *P* = .37).

**Table 3:** Expected benefits of shared medical records

	**Community Health Center Patients** **(n = 295)** **No. (%) in agreement**	**Academic Primary Care Clinic Patients** **(n = 306)** **No. (%) in agreement**	***P* value**	**All Patients** **(N = 601)** **No. (%) in agreement**	**All Physicians** **(N = 564)** **No. (%) in agreement**	***P* value**
Would improve understanding of medical conditions	263 (90)	249 (82)	.01	512 (86)	220 (40)	< .001
Would improve understanding of doctors' instructions	258 (89)	230 (76)	< .001	488 (83)	290 (53)	< .001
Would improve patient adherence	255 (90)	216 (72)	< .001	471 (81)	257 (47)	< .001
Would prepare patients for visits	253 (86)	243 (80)	.04	496 (83)	209 (38)	< .001
Would be reassuring	258 (90)	257 (85)	.06	515 (88)	260 (47)	< .001
Would increase patients' sense of control	263 (91)	252 (83)	.003	515 (87)	388 (70)	.001
Would increase trust in doctors	242 (83)	223 (75)	.02	465 (79)	279 (52)	< .001
Would increase patient satisfaction	254 (89)	244 (82)	.01	498 (85)	240 (44)	< .001
Patients would identify errors in the medical record	231 (83)	253 (85)	.55	484 (84)	177 (32)	< .001
Number of expected benefits, mean (SD)	7.9 (2.0)	7.1 (2.6)	< .001	7.5 (2.3)	4.2 (3.0)	< .001

**Table 4:** Other expectations of shared medical records

	**Community Health** **Center Patients** **(n = 295)** **No. (%) in agreement**	**Academic** **Primary Care** **Clinic Patients** **(n = 306)** **No. (%) in agreement**	***P* value**	**All Patients** **(N = 601)** **No. (%) in agreement**	**All Physicians** **(N = 564)** **No. (%) in agreement**	***P* value**
Lab and x-ray reports would be confusing	146 (50)	109 (36)	< .001	255 (43)	421 (76)	< .001
Doctors' notes would be confusing	130 (44)	84 (28)	< .001	214 (36)	274 (49)	< .001
Would increase patient worry	84 (29)	68 (22)	.07	152 (26)	448 (81)	< .001
Would cause offense or embarrassment	55 (19)	29 (10)	< .001	84 (14)	248 (45)	< .001
Would increase questions between visits	198 (69)	142 (47)	< .001	340 (58)	385 (70)	< .001

### Logistic Model

To assess the determinants of patient attitudes towards Internet-accessible medical records, we created a logistic model. The dependent (outcome) variable was agreement with the statement, “Overall, I think it is a good idea for patients to be able to review their outpatient medical records using the Internet.” Bivariate analyses were performed and demographic variables (listed in Table 1), anticipated benefits, and anticipated concerns that were significant at or below a *P* value of 0.25 were included in the logistic regression. These variables were college graduate (Yes/No), ever used the internet before (Yes/No), anticipating finding doctors' notes confusing (Yes/No), anticipating asking more questions between visits (Yes/No), anticipating being embarrassed or offended (Yes/No), and the number of expected benefits. The variable representing the type of clinic (CHC or academic primary care clinic) the patient was from was also included to account for any difference between the groups. In this model, significant predictors were the following:

1. Previous use of the Internet (OR = 2.45, CI 1.59–3.79)
2. The number of expected benefits (OR = 1.12 per question, CI 1.03–1.21). The mean number of expected benefits for those who endorsed Internet-accessible records was 7.8 vs 7.1 for those who did not endorse them.
3. Anticipating asking more questions between visits (OR = 1.73, CI 1.18–2.54)
4. Anticipating doctors' notes being confusing (OR = 1.50, CI 1.01–2.22)

### Patient Attitudes Compared with Physician Attitudes

The patient responses in aggregate were compared with the responses from the physician survey. Of note, the responses of primary care and specialist physicians were combined, as were responses of patients at the community health centers and the academic clinics, since the differences between patients and physicians was much greater than the differences within physician and patient subgroups. Because the inter-item correlations of the expected benefits was also high among physicians (Cronbach alpha = 0.87), we used the number of expected benefits as for patients.

Physicians were significantly more likely to anticipate concerns than patients (Table 4). Physicians were also significantly less likely to anticipate that shared medical records would be empowering for patients (Table 3).

Physicians were also asked two additional questions about their expectations if patients could routinely review their outpatient medical records. Sixty-three percent anticipated that their “workload would increase substantially,” and 45% anticipated that they “would document things differently in the medical record.”

## Discussion

### Principal Findings in Relation to Previous Studies

This survey confirms the primary results of the surveys in Minnesota [9] and the United Kingdom [10]: the vast majority of patients endorse the concept of patient-accessible medical records, and about half support online access. This survey further demonstrates that these attitudes are shared even by patients in ethnically diverse and socioeconomically disadvantaged populations. On multivariate analysis, demographic features such as age, gender, race, and education did not predict an interest in online patient-accessible records. The primary predictor was previous experience with the Internet, followed by expectations of the benefits and drawbacks of reading the medical record.

Our survey also extends these findings by comparing patient attitudes to the significantly different attitudes of physicians. Patients are particularly likely to anticipate that shared records will be empowering, and particularly unlikely to anticipate that access to their medical records will be embarrassing. Physicians, by contrast, are especially likely to anticipate that laboratory results will confuse patients and that shared records will make patients worry more.

In addition to our quantitative findings, our anecdotal experience in conducting the survey confirmed the strong polarity of opinion towards Internet-accessible records that was reported in the Minnesota survey [9]. After pilot testing our survey for one week, our research assistant was informed by clinic staff that several patients had angrily complained to them after mistakenly inferring that plans were already underway to make their medical records available online. In contrast, in the United Kingdom, where plans *are* underway to give patients online access to their primary care records in 2005, patient attitudes seem to be more favorable, although concerns about security and confidentiality remain [10].

Our results also complement the findings of Hassol et al in the Geisinger Health System in Pennsylvania [4]. In their survey, the experiences of a large group of actual patient users of Geisinger's online health care record were assessed. This system gave patients access to the 25 most frequently ordered laboratory tests with an explanation of the results. This system did not provide access to clinical notes. While less educated patients found test results to be less understandable than higher educated patients, all groups rated understandability as good (71–88 on a scale from 0–100). Although it might be hypothesized that the good general understandability observed in the Geisinger patient group was the result of patient self-selection (patients opting not to use the system if they are concerned about comprehensibility), our own survey suggests that this is not the case. About half of the patients we surveyed in the CHCs, and fewer in the academic primary care clinics, anticipated finding the laboratory and radiographic reports in the medical record to be confusing, but this concern was not a predictor of whether a patient would endorse online shared records. In fact, patients who anticipated finding doctors' notes to be confusing were actually *more* likely to endorse online access. Therefore, the general understandability of Geisinger's health care record is less likely to be the result of self-selection and may be more likely related to other factors (such as the explanations of the test results that were provided by the system).

In addition, Hassol's study reported that Geisinger physicians and system administrators expressed particular concern that patients would be worried about test results that were available online. This information was only anecdotal, however, because their clinician response rate (13%) was too low for statistical analysis. The larger response rate in our statewide physician survey confirms that the majority of physicians are concerned about the potential for shared medical records to confuse or worry their patients.

### Strengths and Limitations of the Study

The different sampling strategies we used for the physician and the patient surveys appear to have been successful in obtaining a representative response of the populations. The response rate for the physician survey is typical of mailed surveys of physicians [14]. The convenience sampling used in the patient survey was successful in recruiting a large sample of ethnically diverse, socioeconomically disadvantaged patients with an excellent response rate. The proportion of patients using the Internet in our sample was comparable to national data from the Pew Internet and American Life project [15]. About half of low-income patients used the Internet, while roughly three quarters of those with higher incomes did. We were surprised, however, by the large proportion of patients (53%) who reported that they had previously reviewed parts of their medical records. This is in sharp contrast to previous reports that only 0.4% of patients spontaneously request their records [16,17] and also to the United Kingdom survey in which only 3.3% of patients reported having seen their records before [10]. We infer that previous surveys assessed whether patients reviewed the full medical record, which few patients have done, while many have reviewed at least part of their medical record. Thus, while patients have limited experience with their medical records, most are not completely naive about the contents.

Several limitations of this study are noted. The attitudes of Colorado physicians and metropolitan Denver patients are only incomplete representations of broader national opinions. Because the patient and the physician surveys were conducted over a year apart, secular changes in attitudes may have affected the comparisons. Also, while the questions in the patient survey and the physician survey were linked, the differences in the way the questions were framed may have accounted for some of the differences observed in the physician and the patient responses.

### Conclusions

Overall, our survey confirms that nearly all patients value having access to their medical records. Clearly, patient-accessible medical records are not something valued only by a privileged elite or by patients with idiosyncratic relationships with the medical system [16]. At the same time, while most patients endorse Internet-accessible records, a substantial minority does not endorse this practice, and many have very strong feelings about it. Presumably, those patients with strong negative feelings are motivated by security and privacy concerns, particularly those without previous experience using the Internet. For Internet-accessible medical records to be more widely adopted, those concerns will need to be thoroughly addressed. Meanwhile, physicians remain more skeptical of the potential benefits of patient-accessible medical records and more sensitive to the potential risks. For physicians to be supportive of programs to increase patients' access to records, the potential benefits of these programs will need to be demonstrated more definitively, and it may be particularly important to address physicians' concerns that these programs may confuse patients or make them anxious. Small trials have suggested that these programs can be implemented without causing harm [2-6]. Larger trials will better define how to enhance the experience of patient-accessible records to promote the benefits that patients expect, and how to mitigate any rare but serious problems that may arise as information from the medical record becomes not only an artifact for medical professionals but a tool for patients as well.

## Multimedia Appendix

Overview of questions asked in physician and patient questionnaires assessing the potential benefits and concerns of sharing medical records.

Actual physician questionnaire.

Actual patient questionnaire.

## Abbreviations

CHC: community health center
OR: odds ratio
