# Supplementary material for: Expectations of Patients and Physicians Regarding Patient-Accessible Medical Records
Source: J Med Internet Res. 2005 May 24;7(2):e13. doi: 10.2196/jmir.7.2.e13 (PMC1550642; doi:10.2196/jmir.7.2.e13)
Supplement: Supplementary file 1 [file jmir_v7i2e13_app1.doc]

# Appendix: Questionnaires for Patients and Physicians

Response options for each question were “strongly disagree,” “disagree,” “agree,” and “strongly agree.” In the physician questionnaire, the questions were framed as, “If you shared outpatient medical records with the adult patients you typically see...” In the patient questionnaire, the questions were framed as, “Even if you have never looked at a medical record before, please indicate how likely it is that the following things would happen if your doctors routinely let you review your outpatient medical records…”

| Question | Patient Survey | Physician Survey |
| --- | --- | --- |
| Lab and x-ray reports would be confusing | I would find the lab and x-ray reports confusing | Your patients would find the lab and x-ray reports confusing |
| Doctors’ notes would be confusing | I would find the doctors’ notes in the medical records confusing | Your patients would find your notes in the medical record confusing |
| Would increase patient worry | I would read things that would make me worry more | Your patients would read things in their records that would make them worry more |
| Would cause offense or embarrassment | I would be embarrassed or offended by some of the tings that my doctors wrote about me | Your patients would be offended by some of the things that are written in the record about them |
| Would increase questions between visits | I would contact my doctors’ offices with more questions between visits | Your patients would contact the practice with more questions between visits |
| Would improve understanding of medical conditions | I would better understand my medical conditions | Your patients would better understand their medical conditions |
| Would improve understanding of doctors’ instructions | I would better understand my doctors’ instructions | Your patients would better understand the instructions you give them |
| Would improve adherence | I would be better at following my doctors’ recommendations | Your patients would be better at following your recommendations |
| Would prepare patients for visits | I would be better prepared for my doctor visits | Your patients would be better prepared for their medical visits with you |
| Would be reassuring | I would feel more reassured | Your patients would feel more reassured |
| Would increase patients’ sense of control | I would feel more in control of my medical care | Your patients would feel more in control of their medical care |
| Would increase trust in doctors | I would trust my doctors more | Your patients would trust you more as their physician |
| Would increase patient satisfaction | I would be more satisfied with my medical care | Your patients would be more satisfied with the medical care you provide |
| Patients would identify errors in the medical record | I would help to identify significant factual errors in the medical record | Your patients would identify significant factual errors in the medical record |
