# Supplementary material for: Expectations of Patients and Physicians Regarding Patient-Accessible Medical Records
Source: J Med Internet Res. 2005 May 24;7(2):e13. doi: 10.2196/jmir.7.2.e13 (PMC1550642; doi:10.2196/jmir.7.2.e13)
Supplement: Supplementary file 2 [file jmir_v7i2e13_app2.doc]

### Patients already have the right to look at their medical records (doctor’s notes, lab tests, x-ray reports, etc.), but most patients never look at their records. Making it easier for patients to look at their records may be helpful in some ways, and may cause problems in others.

### Please indicate below how LIKELY it is that the following things would happen if you shared OUTPATIENT medical records with your adult patients. *Please circle one answer for each question.*

**SD – Strongly DISAGREE**

# D – DISAGREE

# A – AGREE

**SA – Strongly AGREE**

***If you shared outpatient medical records with the adult patients you typically see…***

A1**.** Your patients would find your notes in the

medical record confusing… **SD D A SA**

A2. Your patients would find the lab and x-ray

reports confusing… **SD D A SA**

A3. Your patients would be better prepared for

their medical visits with you…. **SD D A SA**

A4. Your patients would trust you more as their

physician… **SD D A SA**

A5. Your patients would contact the practice with

more questions between visits.. **SD D A SA**

A6. Your patients would read things in their records

that would make them worry more… **SD D A SA**

A7. Your patients would better understand their

medical conditions… **SD D A SA**

A8. Your patients would better understand the

instructions you give them…. **SD D A SA**

A9. Your patients would be offended by some of the

things that are written in the record about them… **SD D A SA**

A10. Your patients would help identify significant

factual errors in the medical record…. **SD D A SA**

A11. Your patients would feel more reassured… **SD D A SA**

A12. Your patients would be better at following your

recommendations…. **SD D A SA**

A13. Your patients would feel more in control of their

medical care… **SD D A SA**

A14. Your patients would be more satisfied with the

medical care you provide… **SD D A SA**

A15. Your workload would increase substantially… **SD D A SA**

A16. You would document things differently in

the medical record… **SD D A SA**

The statements below refer to beliefs that people might have concerning doctors, patients, and medical care. ***For each statement, please check one box indicating how much you agree or disagree.***

| ÿ  Strongly DISAGREE | ÿ  Moderately DISAGREE | ÿ  Slightly DISAGREE | ÿ  Slightly AGREE | ÿ  Moderately AGREE | ÿ  Strongly AGREE |
| --- | --- | --- | --- | --- | --- |

B1. The doctor is the one who should decide what gets talked about during a visit.

| ÿ  Strongly DISAGREE | ÿ  Moderately DISAGREE | ÿ  Slightly DISAGREE | ÿ  Slightly AGREE | ÿ  Moderately AGREE | ÿ  Strongly AGREE |
| --- | --- | --- | --- | --- | --- |

B2. It is often best for patients if they do not have a full explanation of their medical condition.

B3. Patients should rely on their doctor’s knowledge and not try to find out about their conditions on their own.

| ÿ  Strongly DISAGREE | ÿ  Moderately DISAGREE | ÿ  Slightly DISAGREE | ÿ  Slightly AGREE | ÿ  Moderately AGREE | ÿ  Strongly AGREE |
| --- | --- | --- | --- | --- | --- |

B4. Many patients continue asking questions even though they are not learning anything new.

| ÿ  Strongly DISAGREE | ÿ  Moderately DISAGREE | ÿ  Slightly DISAGREE | ÿ  Slightly AGREE | ÿ  Moderately AGREE | ÿ  Strongly AGREE |
| --- | --- | --- | --- | --- | --- |

| ÿ  Strongly DISAGREE | ÿ  Moderately DISAGREE | ÿ  Slightly DISAGREE | ÿ  Slightly AGREE | ÿ  Moderately AGREE | ÿ  Strongly AGREE |
| --- | --- | --- | --- | --- | --- |

B5. Patients should be treated as if they were partners with the doctor, equal in power and status.

| ÿ  Strongly DISAGREE | ÿ  Moderately DISAGREE | ÿ  Slightly DISAGREE | ÿ  Slightly AGREE | ÿ  Moderately AGREE | ÿ  Strongly AGREE |
| --- | --- | --- | --- | --- | --- |

B6**.** Patients generally want reassurance rather than information about their health.

B7. When patients disagree with their doctor, this is a sign that the doctor does not have the patient’s respect and trust.

| ÿ  Strongly DISAGREE | ÿ  Moderately DISAGREE | ÿ  Slightly DISAGREE | ÿ  Slightly AGREE | ÿ  Moderately AGREE | ÿ  Strongly AGREE |
| --- | --- | --- | --- | --- | --- |

B8. The patient must always be aware that the doctor is in charge.

| ÿ  Strongly DISAGREE | ÿ  Moderately DISAGREE | ÿ  Slightly DISAGREE | ÿ  Slightly AGREE | ÿ  Moderately AGREE | ÿ  Strongly AGREE |
| --- | --- | --- | --- | --- | --- |

B9. When patients look up medical information on their own, this usually confuses more than it helps.

| ÿ  Strongly DISAGREE | ÿ  Moderately DISAGREE | ÿ  Slightly DISAGREE | ÿ  Slightly AGREE | ÿ  Moderately AGREE | ÿ  Strongly AGREE |
| --- | --- | --- | --- | --- | --- |

The questions below refer to your practice setting and current office practices. ***For each question, please circle one answer.***

C1. Do you see patients in an office setting?   **YES   NO**

C2.  Do you already routinely send copies of your clinical notes to your patients?  **YES  NO**
