# Supplementary material for: Expectations of Patients and Physicians Regarding Patient-Accessible Medical Records
Source: J Med Internet Res. 2005 May 24;7(2):e13. doi: 10.2196/jmir.7.2.e13 (PMC1550642; doi:10.2196/jmir.7.2.e13)
Supplement: Supplementary file 3 [file jmir_v7i2e13_app3.doc]

### Survey: Patients’ Interests in the Medical Record

### Patients already have the right to look at their medical records (doctor’s notes, lab tests, x-ray reports, etc.), but most patients never look at their records. Making it easier for patients to look at their records may be helpful in some ways, and may cause problems in others. We are interested in how you would feel about looking at your own medical records.

### Thank you for completing this survey. It will take about 15 minutes to complete. Your name will not be placed on the survey, and your answers will remain confidential. When you are finished with the survey, please fold it in half and place it in the box provided. You may tear off this cover sheet for your records if you like.

### If you have any questions about this survey, you may ask the research assistant who gave you the survey. You may also contact the Principal Investigator of the study:

### Dr. Stephen Ross

### 1635 N. Ursula Street, Mailstop F729

### Aurora, Colorado 80045

### (303) 372-9088

### Subject Number

### Even if you have never looked at a medical record before, please indicate below how LIKELY it is that the following things would happen if your doctors routinely let you review your outpatient medical records.

### *Please check one answer for each question.*

###### If my doctors routinely shared my outpatient medical records with me…

| A1. I would find the doctors’ notes in the medical records confusing. |  |   Strongly DISAGREE |  DISAGREE |  AGREE |   Strongly AGREE |
| --- | --- | --- | --- | --- | --- |
| A2. I would find the lab and x-ray reports confusing. |  |   Strongly DISAGREE |  DISAGREE |  AGREE |   Strongly AGREE |
| A3. I would be better prepared for my doctor visits. |  |   Strongly DISAGREE |  DISAGREE |  AGREE |   Strongly AGREE |
| A4. I would trust my doctors more. |  |   Strongly DISAGREE |  DISAGREE |  AGREE |   Strongly AGREE |
| A5. I would contact my doctors’ offices with more questions between visits. |  |   Strongly DISAGREE |  DISAGREE |  AGREE |   Strongly AGREE |
| A6. I would read things that would make me worry more. |  |   Strongly DISAGREE |  DISAGREE |  AGREE |   Strongly AGREE |
| A7. I would better understand my medical conditions. |  |   Strongly DISAGREE |  DISAGREE |  AGREE |   Strongly AGREE |

***Please continue to the next page.***

| A8. I would better understand my doctors’ instructions. |  |   Strongly DISAGREE |  DISAGREE |  AGREE |   Strongly AGREE |
| --- | --- | --- | --- | --- | --- |
| A9. I would be embarrassed or offended by some of the things that my doctors wrote about me. |  |   Strongly DISAGREE |  DISAGREE |  AGREE |   Strongly AGREE |
| A10. I would help to identify significant factual errors in the medical record. |  |   Strongly DISAGREE |  DISAGREE |  AGREE |   Strongly AGREE |
| A11. I would feel more reassured. |  |   Strongly DISAGREE |  DISAGREE |  AGREE |   Strongly AGREE |
| A12. I would be better at following my doctors’ recommendations. |  |   Strongly DISAGREE |  DISAGREE |  AGREE |   Strongly AGREE |
| A13. I would feel more in control of my medical care. |  |   Strongly DISAGREE |  DISAGREE |  AGREE |   Strongly AGREE |
| A14. I would be more satisfied with my medical care. |  |   Strongly DISAGREE |  DISAGREE |  AGREE |   Strongly AGREE |
| A15. OVERALL, I think it is a good idea for patients to be able to routinely review their outpatient medical records. |  |   Strongly DISAGREE |  DISAGREE |  AGREE |   Strongly AGREE |
| A16. I would be interested in reviewing my own outpatient medical records. |  |   Strongly DISAGREE |  DISAGREE |  AGREE |   Strongly AGREE |
| A17. OVERALL, I think it is a good idea for patients to be able to review their outpatient medical records using the Internet. |  |   Strongly DISAGREE |  DISAGREE |  AGREE |   Strongly AGREE |
| A18. I would be interested in reviewing my own medical records using the Internet. |  |   Strongly DISAGREE |  DISAGREE |  AGREE |   Strongly AGREE |

***Please continue to the next page.***

The statements below refer to beliefs that people might have concerning doctors, patients, and medical care.

***For each statement, please check one box indicating how much you agree or disagree.***

| B1. The doctor is the one who should decide what gets talked about during a visit. |  |   Strongly  DISAGREE |   Moderately  DISAGREE |   Slightly  DISAGREE |   Slightly  AGREE |   Moderately  AGREE |   Strongly  AGREE |
| --- | --- | --- | --- | --- | --- | --- | --- |
| B2. It is often best for patients if they do not have a full explanation of their medical condition. |  |   Strongly  DISAGREE |   Moderately  DISAGREE |   Slightly  DISAGREE |   Slightly  AGREE |   Moderately  AGREE |   Strongly  AGREE |
| B3. Patients should rely on their doctor’s knowledge and not try to find out about their conditions on their own. |  |   Strongly  DISAGREE |   Moderately  DISAGREE |   Slightly  DISAGREE |   Slightly  AGREE |   Moderately  AGREE |   Strongly  AGREE |
| B4. Many patients continue asking questions even though they are not learning anything new. |  |   Strongly  DISAGREE |   Moderately  DISAGREE |   Slightly  DISAGREE |   Slightly  AGREE |   Moderately  AGREE |   Strongly  AGREE |
| B5. Patients should be treated as if they were partners with the doctor, equal in power and status. |  |   Strongly  DISAGREE |   Moderately  DISAGREE |   Slightly  DISAGREE |   Slightly  AGREE |   Moderately  AGREE |   Strongly  AGREE |
| B6. Patients generally want reassurance rather than information about their health. |  |   Strongly  DISAGREE |   Moderately  DISAGREE |   Slightly  DISAGREE |   Slightly  AGREE |   Moderately  AGREE |   Strongly  AGREE |
| B7. When patients disagree with their doctor, this is a sign that the doctor does not have the patient’s respect and trust. |  |   Strongly  DISAGREE |   Moderately  DISAGREE |   Slightly  DISAGREE |   Slightly  AGREE |   Moderately  AGREE |   Strongly  AGREE |

***Please continue to the next page.***

| B8. The patient must always be aware that the doctor is in charge. |  |   Strongly  DISAGREE |   Moderately  DISAGREE |   Slightly  DISAGREE |   Slightly  AGREE |   Moderately  AGREE |   Strongly  AGREE |
| --- | --- | --- | --- | --- | --- | --- | --- |
| B9. When patients look up medical information on their own, this usually confuses more than it helps. |  |   Strongly  DISAGREE |   Moderately  DISAGREE |   Slightly  DISAGREE |   Slightly  AGREE |   Moderately  AGREE |   Strongly  AGREE |

The questions below ask about your interest in getting extra information about how well your doctor practices medicine.

***For each question, please check one box indicating how much you agree or disagree.***

| C1. Suppose your usual doctor was treating you for years for a condition like diabetes or high blood pressure. How useful would it be for you to receive information on your usual doctor’s success in treating that condition? |  |   NOT AT ALL useful |   SLIGHTLY useful |   MODERATELY useful |   QUITE A BIT useful |   EXTREMELY useful |
| --- | --- | --- | --- | --- | --- | --- |
| C2. Suppose you needed to see a surgeon to set up a major operation. How useful would it be for you to receive information on that surgeon’s success in treating your condition? |  |   NOT AT ALL useful |   SLIGHTLY useful |   MODERATELY useful |   QUITE A BIT useful |   EXTREMELY useful |
| C3. Suppose that you needed to see a heart specialist for treatment of a serious heart problem. How useful would it be for you to receive information on that heart specialist’s success in treating your condition? |  |   NOT AT ALL useful |   SLIGHTLY useful |   MODERATELY useful |   QUITE A BIT useful |   EXTREMELY useful |

***Please continue to the next page.***

| C4. If you were to be given information about a doctor’s success in treatment, would you like information in the form of a letter grade (such as “A-” or “B”)? |  |   Yes |   No |  |  |  |
| --- | --- | --- | --- | --- | --- | --- |
| C5. If you were to be given information about a doctor’s success in treatment, would you like to see numbers or statistics (such as “68% of patients who get surgery have complete relief of pain”)? |  |   Yes |   No |  |  |  |
| C6. If you were to be given information about a doctor’s success in treatment, would you like to see how this doctor compares to other doctors? |  |   Yes |   No |  |  |  |

Patients with different backgrounds may have different attitudes about reading their medical records. Please provide the following information on your background. All of this information will be kept completely confidential.

D1. Please write in the last two digits of the year you were born. (NOTE: If you were born before 1915, please write in “XX” rather than your birth year):

| **1** | **9** |  |  |
| --- | --- | --- | --- |

***Please continue to the next page.***

***For questions C1-C8, please check one box:***

| D2. Are you male or female? |   Male |   Female |  |  |  |  |  |
| --- | --- | --- | --- | --- | --- | --- | --- |
| D3. What is the highest grade or year of school you completed? |   Grades 1-8 |   Grades 9-11 (Some high school) |   High school graduate |   Some college |   College graduate |   Post-graduate education |  |
| D4. Have you reviewed parts of your medical records before? |   Yes |   No |  |  |  |  |  |
| D5. Have you ever used the Internet before? |   Yes |   No |  |  |  |  |  |
| D6. Do you have access to the Internet at home or at work? |   Yes |   No |  |  |  |  |  |
| D7. Have you used e-mail in the last month? |   Yes |   No |  |  |  |  |  |
| D8. Would you be interested in communicating with your doctor using e-mail? |   Yes |   No |  |  |  |  |  |
| D9. How often do you usually see a doctor (including both your primary care doctor and specialists)? |   Less than once a year |   About once a year |   2 or 3 times a year |   4 or 5 times a year |   6 or more times a year |  |  |
| D10. What is you household income per year? |   Less than $15,000 per year |   $15-29,000 |   $30-44,000 |   $45-59,000 |   $60-74,000 |   $75-90,000 |   over $90,000 |

***Please continue to the next page.***

***For question C9 and C10, please indicate with a check mark every answer that applies:***

D11. How would your describe your race or ethnic background?

|  | **White / Caucasian** |
| --- | --- |
|  | **Hispanic** |
|  | **Black / African-American** |
|  | **Asian / Pacific Islander** |
|  | **Native American** |
|  | **Other : Please specify** |

D12. Please indicate what forms of medical insurance coverage you have:

|  | **Medical insurance sponsored by an employer** |
| --- | --- |
|  | **Medical insurance purchased outside of an employer** |
|  | **Military / VA / TriCare / CHAMPUS / ChampVA** |
|  | **Medicare** |
|  | **Medicaid** |
|  | **Formal discounted medical program, such as the Colorado Indigent Care Program (CICP)** |
|  | **No insurance** |

***Thank you for completing the survey!***

***We would appreciate any comments you may have about reading your medical records, or about this survey:***
